# Concerns Regarding Renal Function Claims in Low‐Dose Spironolactone Study

**DOI:** 10.1002/edm2.70085

**Published:** 2025-07-19

**Authors:** Özant Helvacı, Burçak Cavnar Helvacı

**Affiliations:** ^1^ Department of Nephrology Gazi University Faculty of Medicine Ankara Turkey; ^2^ Department of Endocrinology and Metabolism Ankara Etlik City Hospital Ankara Turkey

Dear Editor,

We read with interest the study by Amini et al. on low‐dose spironolactone in diabetic kidney disease [1]. While we appreciate the authors' investigation of safety at reduced dosing, we believe some aspects of their eGFR interpretation need clarification from a nephrology perspective.

The authors report a statistically significant increase in eGFR from 71.83 ± 19.09 to 74.25 ± 17.81 mL/min/1.73 m^2^ (*p* = 0.042) and describe this as renal function improvement. This interpretation warrants careful consideration:

First, creatinine measurement has inherent analytical variability, and small changes in calculated eGFR may not reflect true kidney function changes. The magnitude of change (3.4%) falls within the expected coefficient of variation for creatinine‐based eGFR calculations. A systematic review and meta‐analysis of 37 studies with 2770 participants demonstrate that eGFR fluctuations of 5.0%–5.2% likely represent analytical noise rather than clinically meaningful change [2]. Such small variations should be interpreted cautiously without confirmatory measurements.

Second, we note that the concurrent decrease in serum creatinine (1.08 ± 0.23 to 1.01 ± 0.23 mg/dL) is difficult to explain mechanistically, as spironolactone does not enhance creatinine clearance or reduce creatinine generation. Furthermore, mineralocorticoid receptor antagonists characteristically reduce GFR through hemodynamic effects, including afferent arteriole vasoconstriction and decreased glomerular filtration pressure [3]. An increase in eGFR contradicts established pharmacological mechanisms.

Third, previous studies with identical 12.5 mg spironolactone dosing have consistently shown stable or slightly decreased eGFR, not increases. For example, Oiwa et al.'s multicenter randomised controlled trial demonstrated that eGFR remained stable from 64.2 ± 17.6 to 60.2 ± 16.1 mL/min/1.73 m^2^ in the spironolactone group while achieving significant albuminuria reduction [4]. This rigorous randomised controlled trial design with proper control group provides higher‐quality evidence than the current pre‐post intervention study. The present findings are inconsistent with this established pattern from controlled trials.

Finally, recent evidence from the FIDELITY pooled analysis of finerenone trials demonstrates that cardiovascular and kidney benefits were maintained regardless of acute eGFR changes at month 1, with similar efficacy across patients experiencing > 10% eGFR decline, stable eGFR, or eGFR increases, confirming that early eGFR changes do not correlate with long‐term hard outcomes [5]. We suggest focusing on clinically meaningful endpoints rather than over‐interpreting short‐term eGFR fluctuations.

We recommend the authors consider adopting more conservative language regarding renal function changes and acknowledge the limitations of short‐term eGFR monitoring. Future studies would benefit from incorporating confirmatory measurements, longer follow‐up periods and mechanistic assessments to better characterise true renal function changes with low‐dose spironolactone therapy.

## Author Contributions

**Özant Helvacı:** conceptualization, writing – original draft. **Burçak Cavnar Helvacı:** writing – review and editing, conceptualization.

## Conflicts of Interest

The authors declare no conflicts of interest.

## Data Availability

The authors have nothing to report.
